# Randomized, multicentre assessment of the efficacy and safety of ASAQ – a fixed-dose artesunate-amodiaquine combination therapy in the treatment of uncomplicated *Plasmodium falciparum *malaria

**DOI:** 10.1186/1475-2875-8-125

**Published:** 2009-06-08

**Authors:** Jean Louis Ndiaye, Milijaona Randrianarivelojosia, Issaka Sagara, Philippe Brasseur, Ibrahima Ndiaye, Babacar Faye, Laurence Randrianasolo, Arsène Ratsimbasoa, Doris Forlemu, Vicky Ama Moor, Aminata Traore, Yahia Dicko, Niawanlou Dara, Valérie Lameyre, Mouctar Diallo, Abdoulaye Djimde, Albert Same-Ekobo, Oumar Gaye

**Affiliations:** 1 Department of Parasitology, Université Cheikh Anta Diop, Dakar, Senegal; 2 Unité de Recherche sur le Paludisme, Institut Pasteur de Madagascar, Antananarivo, Madagascar; 3 Malaria Research and Training Center, Department of Epidemiology of Parasitic Diseases, Faculty of Medicine, Pharmacy and Odonto-Stomatology, University of Bamako, Bamako, Mali; 4 Institut de Recherche pour le Développement, Dakar, Senegal; 5 Faculté de Médecine et des Sciences Biomédicales, Centre Hospitalo-Universitaire Yaounde, Cameroon; 6 Access to Medicines, sanofi-aventis, Paris, France

## Abstract

**Background:**

The use of artemisinin derivative-based combination therapy (ACT) such as artesunate plus amodiaquine is currently recommended for the treatment of uncomplicated *Plasmodium falciparum *malaria. Fixed-dose combinations are more adapted to patients than regimens involving multiple tablets and improve treatment compliance. A fixed-dose combination of artesunate + amodiaquine (ASAQ) was recently developed. To assess the efficacy and safety of this new combination and to define its optimum dosage regimen (once or twice daily) in the treatment of uncomplicated *P. falciparum *malaria, a multicentre clinical study was conducted.

**Methods:**

A multicentre, randomized, controlled, investigator-blinded, parallel-group study was conducted in five African centers in Cameroon, Madagascar, Mali and Senegal from March to December 2006. Efficacy and safety of ASAQ were assessed compared to those of artemether + lumefantrine (AL). The WHO protocol with a 28-day follow-up for assessing the drug therapeutic efficacy was used. Patients suffering from uncomplicated *P. falciparum *malaria were randomized to receive ASAQ orally once daily (ASAQ1), ASAQ twice daily (ASAQ2) or AL twice daily (AL) for three days. The primary outcome was PCR-corrected parasitological cure rate and clinical response.

**Results:**

Of 941 patients initially randomized and stratified into two age groups (<5 years, and ≥5 years), 936 (99.5%) were retained for the intent to treat (ITT) analysis, and 859 (91.3%) patients for the per protocol (PP) analysis. Among ITT population, up to D28, PCR-corrected adequate parasitological and clinical response rates were 95.2% in the ASAQ1 group, 94.9% in the ASAQ2 group and 95.5% in the AL group. Moreover, the cure rate evaluated among PP population was ≥98.5% in both ASAQ therapeutic arms. Therapeutic response rates did not display any significant differences between age groups or between one geographical site and another. Altogether, this demonstrates the non-inferiority of ASAQ1 regimen compared to both ASAQ2 and AL regimens. During follow-up mild and moderate adverse events including gastrointestinal and/or nervous disorders were reported in 29.3% of patients, with no difference between groups in the nature, frequency or intensity of adverse events.

**Conclusion:**

The non-inferiority of ASAQ compared with AL was demonstrated. The fixed-dose combination artesunate + amodiaquine (ASAQ) is safe and efficacious even in young children under 5 years of age. Whilst administration on a twice-a-day basis does not improve the efficacy of ASAQ significantly, a once-a-day intake of this new combination clearly appears as an effective and safe therapy in the treatment of uncomplicated *P. falciparum *malaria both in adults and children. Implications of such findings are of primary importance in terms of public health especially in African countries. As most national policies plan to strengthen malaria control to reach the elimination of this disease, anti-malarial drugs such as the artesunate + amodiaquine fixed-dose ACT will play a pivotal role in this process.

**Trial registration:**

The protocol was registered with the www.clinicaltrials.gov open clinical trial registry under the identifier number NCT00316329.

## Background

Combination therapy has been on the path to become the treatment of choice for uncomplicated *Plasmodium falciparum *malaria or the past decade [1]. The use of oral artemisinin-based combination therapy (ACT) combining an artemisinin derivative with anti-malarial agents with a long half-life is particularly encouraged. Randomized clinical trials have previously demonstrated that the combination of artesunate (AS) with amodiaquine (AQ) could improve cure rates and reduce gametocytemia more efficiently than a monotherapy with AQ alone [2]. Since this (AS) + (AQ) combination proved well-tolerated too, a co-blister containing AS and AQ (such as Arsucam^®^) was designed and marketed in 2002. The results of different clinical trials confirmed the efficacy of Arsucam^® ^in Senegal [3,4], the Comoros Islands [5], Mali [6] and Cameroon [4]. The efficacy of the AS + AQ combination was confirmed by routine use as part of national malaria treatment policy [7].

The 2006 WHO guidelines for malaria treatment recommend the combination of AS and AQ in a ratio of 2.5 (corresponding to the theoretical dosage of both drugs used in monotherapy) or 3.1 (corresponding to the co-blister's ratio). However, the use of AS and AQ in multiple tablet combinations is unsatisfactory in terms of treatment compliance. It involves the risk that only one of the two drugs be received or that the drugs be administered at an inadequate dose. Since it is recognized in the medical fields that the availability of single-tablet formulations (containing drugs in a fixed-dose combination) reduces these risks and improves treatment compliance [8-10], such a combination was developed by a public-private partnership between DNDi and sanofi-aventis, to provide AS and AQ in a single tablet (ASAQ). A 1:2.7 dose-ratio of AS:AQ was determined according to the accepted therapeutic doses of the components individually. Finally, the dose regimen was stratified by weight and age to ensure optimal doses delivery [11]. In a first pivotal Phase III study performed in Burkina Faso ASAQ showed non-inferiority to the combination of AS and AQ in children with *P. falciparum *infections [12]. In order to complete the phase III program before WHO prequalification, a second multicentre study was initiated, whose results are reported herein. The objective of this study was to assess the efficacy and safety of ASAQ fixed-dose combination tablets in comparison with a fixed-dose combination of artemether + lumefantrine (AL), then to define the optimum dosage regimen of ASAQ (1 or 2 daily intakes per day) in the treatment of uncomplicated *P. falciparum *malaria.

## Methods

### Study design

This was a multicentre, randomized, controlled, investigator-blinded, parallel-group study comparing three ACT regimens in patients with uncomplicated *P. falciparum *malaria. The study was conducted in five African sites where malaria transmission is perennial: Bancoumana (Mali), Yaoundé (Cameroon), Tsiroanomandidy (Madagascar), Keur-Socé and Mlomp (Senegal). Patients were included between March and December 2006. The study was conducted during the highest malaria transmission period for each site. This protocol was registered with the www.clinicaltrials.gov open clinical trial registry under the identifier number NCT00316329.

### Patients

The study included patients infected with *P. falciparum *residing in the study area throughout the planned period and according to the following criteria: weighing ≥ 10 kg; displaying a parasitaemia from 1,000 to 200,000 asexual forms per microliter of blood; presenting an axillary temperature ≥ 37.5°C or suffering from fever within the last 24 hours and capable of receiving oral treatment.

Patients with any clinical sign of severe malaria [13] or any serious concomitant disease, such as cardiovascular disease were excluded. Similarly exclusion was applied in case of concomitant intake of medications involving cytochrome P450 2D6 (CYP2D6) pathway and in case of documented intolerance to any medications used in this study. Pregnant or breast-feeding women were also excluded and a urine pregnancy test was performed on all women of child-bearing age. Successive enrolments in this study or simultaneously in any other clinical trial were also prohibited.

### Randomization and treatment

Patients were randomized to one of three treatment groups, namely ASAQ bi-layer fixed-dose combination tablets once daily intake (ASAQ1), ASAQ twice daily (ASAQ2), and AL twice daily (AL). Randomization was stratified according to patient age using separate randomization lists. The two age strata were children under five years of age (as the primary WHO target population) and patients aged five years and over. Treatment dosages were determined according to patient's body weight. Treatment duration was three days. An eight hour time-lapse was requested in case of twice-daily regimens (usually the morning and evening doses). Tablets were orally administered with a small amount of drinking water; and patients were advised to resume a normal diet as soon as possible. All patients were monitored for 30 minutes after administration in order to ensure that the drug was not lost after an eventual vomiting episode. When this occurred, the same dose was re-administered. If vomiting re-occured, the subject was withdrawn from the study and a replacement treatment with another effective anti-malarial, generally quinine, was provided. In order to ensure blinding, all patients in the same body weight range received the same number of tablets per intake, which was defined by the recommended dose of AL. Patients in the ASAQ arms were given placebo tablets to match this number. Only the person administering the treatment was aware of the nature of the drug taken by each participant, whereas investigators evaluating safety and efficacy were kept blind.

### Efficacy and safety assessment

The WHO protocol with a 28-day follow-up [14] to assess the anti-malarial drug therapeutic efficacy was used (Table 1). Follow-up visits were systematically performed on Days 1, 2, 3, 7, 14, 21 and 28 after enrolment. Each visit consisted of a physical examination combining evaluation of clinical safety and measurement of vital signs. Axillary temperature was measured using an electronic thermometer. Blood pressure and pulse were measured after a 10-minutes rest in sitting position. Blood samples were collected on Days 0, 7 and 28 for parasitology (parasites count), haematology (leucocytes and platelets count; haemoglobin level) and biochemistry (blood glucose, creatinine, alanine aminotransferase ALT, aspartate aminotransferase AST). Blood samples were exceptionally collected once more on Day 14 if abnormal results were observed on Day 7.

**Table 1:** Investigational plan

STUDY PARAMETERS	REFERENCE DAYS								Unplanned visits
		
	D0	D1	D2	D3	D7 ± 1d	D14 ± 1d	D21 ± 1d	D28 ± 1d	
History	X								
Age/Weight/Height/sex	X								
Physical examination/Vital signs	X	X	X	X	X	X	X	X	X
Clinical safety	X	X	X	X	X	X	X	X	X
Parasitological examination	X	X	X	X	X	X	X	X	X
Haemoglobin^a^	X				X	X^b^		X	(X)
Platelets + leukocytes	X				X	X^b^		X	(X)
Blood glucose^a^	X								(X)
Blood creatinine	X				X	X^b^		X	(X)
AST/ALT	X				X	X^b^		X	(X)
Preparation of filter paper blood spot samples for PCR	X				X	X	X	X	(X)
Study treatment	X	X	X						
Concomitant medication	X	X	X	X	X	X	X	X	(X)

^a ^in the event of onset of severe indicators; ^b ^in the event of an abnormal value on D7; (x) if necessary.

Clinical efficacy was assessed by grading the pre-existing clinical signs and combining them with the temperature values. Parasitological efficacy was based on asexual parasitaemia, although a gametocyte count was also systematically performed. The following clinical symptoms were assessed systematically at each visit and graded as absent, mild, moderate, severe or very severe: perspiration, headache, chills, pain (specifying topography), jaundice, asthenia, dizziness, anorexia, skin fold, skin rash, hepatomegaly, pruritus. Splenomegaly was estimated according to the Hackett scale [15]. Vomiting and stools were quantified and the presence of diarrhea investigated. The primary endpoint was the rate of adequate parasitological and clinical response after PCR correction on Day 28, following the 2003 *in vivo *WHO protocol. Clinical safety was monitored through regular patient interviews with regards to the occurrence of adverse events following the previous visit.

### Laboratory analyses

Fingerpick blood samples were collected for parasitological checking at enrolment and at successive follow-up visits. Thin and thick smears were obtained and stained with May Grünwald-Giemsa. Filter paper dried blood spot samples were retained on filter paper for later PCR analysis in case of recurrent parasitaemia. Blood samples for parasitological count were analyzed at local centers under their own procedures. Giemsa-stained thick blood smears were read by experienced microscopy practitioners who were blinded to treatment allocation. Parasite densities were calculated by counting the number of asexual and sexual *P. falciparum *parasites until 300 leukocytes were observed and then converting this figure into parasites (trophozoits and/or gametocytes) per microliter of blood, assuming an average leukocyte count of 7,500/μL. For quality control purposes, a random sample of at least 10% of all slides analyzed at each site, as well as all slides detected as positive, were re-evaluated using a blinded procedure with respect to the original result and to the source study center at the Malaria Research and Training Center (MRTC), Bamako, Mali.

### PCR analysis

For participants with recurrent parasitemia after day 7, paired polymerase chain reaction (PCR) blots (from day 0 and the day of parasitemia recurrence) were analyzed for parasite merozoite surface proteins (MSP-1 and MSP-2) and microsatellite CA1 to distinguish between reinfection and recrudescence [16]. DNA was obtained from Day 0 and failure day samples. Initial DNA was extracted using the methanol method. Samples that failed to yield interpretable results were re-extracted using a Qiagen Kit (Qiagen, Valencia, CA) according to the manufacturer's instructions. Day 0 and failure day alleles of *msp*-1, *msp*-2, and microsatellite *Ca*1 gene loci were compared. The PCR was performed using the following primers pairs MSP1: O1 = 5'-CACATGAAAGTTATCAAGAACTTGTC-3' and O2 = 5'-GTACGTCTAATTCATTTGCACG-3' for PCR1, N1 = 5'-GCAGTATTGACAGGTTATGG-3' and N2 = 5'-GATTGAAAGGTATTTGAC-3' for PCR2; MSP2: S3 = 5'-GAAGGTAATTAAAACATTGTC-3' and S2 = 5'-GAGGGATGTTGCTGCTCCACAG-3' for PCR1, S1 = 5'-GAGTATAAGGAGAAGTATG-3' and S4 = 5'-CTAGAACCATGCATATGTCC-3' for PCR2; and MICROSATELLITE Ca1: Ca1-1L = 5'-GCTGTAAAACGTGAACAAAAA-3 and Ca1-1R = 5'-CAATTCTGCTTCAGTTGGATT-3' for PCR1 and Ca1-L = 5'-ATTATGAACAATTCAGAC-3' and Ca1-R = 5'-GTTGTTATAGCTAATGAG-3' for PCR2. One to five microliters of DNA was amplified for 30 cycles during the primary PCR.

One to two microliters of the product of that amplification was used for another 30 cycles of PCR. Each PCR was performed with 1 μM of each primer, 200 μM of each dNTP, and 1.5–3 mM MgCl2.

Possible outcomes were i) recrudescence, if the alleles of the pre-treatment and post-treatment samples were the same for *msp*-1, *msp*-2, and Ca1; ii) reinfection, if the alleles of the pre-treatment and post-treatment samples were distinct for any of these three loci; iii) mixed recrudescence and reinfection, if similar alleles were found in the pre-treatment and posttreatment samples for all the markers as mentioned above, but with additional distinct alleles identified; and iv) indeterminate, if either or both the pre-treatment and post-treatment samples could not be amplified. Mixed recrudescent and re-infection cases were computed as recrudescent.

### Sample size calculation

*A priori *power calculations were used to determine the sample size required to demonstrate non-inferiority between the ASAQ group and the AL group (inter-group difference <5%) in both the overall population and in a target subpopulation of children under five years of age with a type I error of 5% and a power of 80% to be able to detect such a difference. On the basis of previous studies with AL [17,18], a response rate of 97% for children under five years of age and 98% for older patients was anticipated. The sample size calculation estimate led to the assumption that 174 children under five and 138 older patients in each treatment arm would be required, making a total target sample size of 936. Three patient populations were evaluated. The safety population comprised all patients who received at least one dose of treatment. The intent to treat (ITT) population comprised all patients in the safety population, with the exception of those who had rejected the treatment twice by vomiting following the first administration. The per-protocol (PP) population comprised all patients of the ITT population with the exception of those presenting a major protocol deviation.

### Statistical analysis

The primary efficacy endpoint corresponded to parasitological and clinical cure on Day 28 determined in the ITT population. The difference in the proportions of responders between the ASAQ group and AL group was estimated using a non-inferiority analysis with respect to a 5% significance level, according to the Binomial distribution, using the STATXACT software program with the "noninf" option (non-inferiority delta of 5%). Categorical variables were compared with the χ^2 ^test or Fisher's exact test, normally distributed quantitative variables with Student's *t*-test and other quantitative variables with the Wilcoxon test. Missing data were not replaced. All statistical analyses were performed centrally using SAS version 8.2 software (SAS Institute, Cary, North Carolina, USA).

### Ethical issues

This study was conducted according to the Declaration of Helsinki and existing national legal and regulatory requirements. The protocol was submitted and approved by the appropriate ethics committees and institutional review boards in each participating country. Written informed consent was obtained from each participant or their parent or guardian in case of minors. If patients were unable to sign, a fingerprint was applied on the consent form. In case of patients unable to read, the information was read and explained to them in the appropriate language. In such cases, the presence of a witness who also signed the consent form to confirm that the patient had freely given consent was required.

## Results

**Figure 1:**
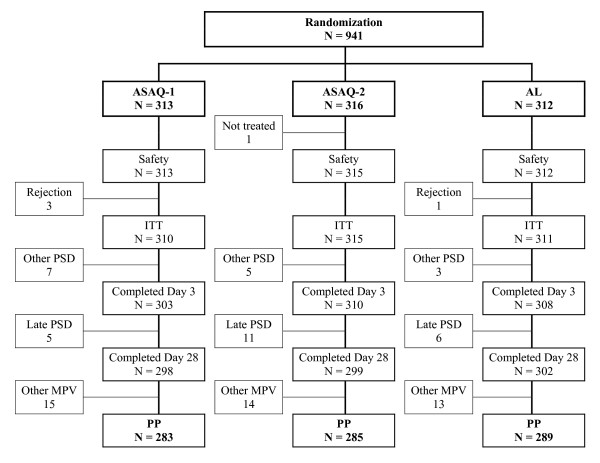
**Patient flow through the study**. AS: artesunate; AQ: amodiaquine; A: artemether; L: lumefantrine; ITT: intent to treat; PP: per protocol; MPV: major protocol violation; PSD: premature study discontinuation.

Overall, 941 patients were randomized to be included in one of the three therapeutic arms. Since one patient (0.1%) did not receive any study medication, the safety population consisted of 940 patients. Table 2 displays patient features at enrolment time. Four patients (0.4%) were excluded on Day 0 after repeated rejection of their first drug intake. The flow of patients through the study is displayed in Figure 1. No significant differences in the frequency or nature of premature study discontinuations were observed between treatment groups, or in the frequency or the nature of major protocol deviations. On Day 28, the per-protocol population consisted of 857 patients. Globally, frequency and severity of signs and symptoms of malaria were equivalent between the three treatment groups and between the two age-group strata (Table 3).

**Table 2:** Characteristics of patients in the safety population at inclusion.

	ASAQ1 group **(N = 313)**	ASAQ2 group **(N = 315)**	AL group **(N = 312)**
Age (years)			
Mean ± SD	9.5 ± 10.8	9.1 ± 9.8	9.4 ± 10.7
< 5 years	146 (46.6)	148 (47.0)	142 (45.5)
≥ 5 years	167 (53.4)	167 (53.0)	170 (54.5)
Gender			
Male	176 (56.2)	170 (54.0)	167 (53.5)
Female	137 (43.8)	145 (46.0)	145 (46.5)
Weight (kg)	24.8 ± 18.8	24.6 ± 17.7	24.6 ± 17.4
Parasite density (/μL)	37780.9 ± 45651.6	41863.0 ± 49049.1	33958.3 ± 41473.5
Gametocyte density (/μL)*	173.1 ± 296.5 (n = 14)	256.0 ± 317.3 (n = 14)	376.0 ± 1037.2 (n = 15)

ASAQ1: ASAQ 1 daily intake; ASAQ2: ASAQ 2 daily intakes; AL: AL 2 daily intakes

Data are presented as mean ± SD for quantitative variables and as patient numbers (%) for categorical variables. *Information on gametocyte density is only provided for the small number of subjects (N = 43 overall) with gametocytaemia at inclusion.

**Table 3:** Clinical characteristics at inclusion in the safety population.

	ASAQ1 group (N = 313)	ASAQ2 group (N = 315)	AL group (N = 312)
Axillary temperature (°C)	37.94 ± 1.09	38.04 ± 1.04	37.92 ± 1.07
Asthenia	272 (86.9%)	277 (87.9%)	270 (86.5%)
Anorexia	260 (83.1%)	253 (80.3%)	250 (80.1%)
Headache*	172/212 (81.1%)	191/231 82.7%)	181/219 (82.6%)
Chills	150 (47.9%)	156 (49.5%)	147 (47.1%)
Perspiration	146 (46.6%)	153 180 (57.1%)	147 183 (58.7%)
Dizziness*	53/165 (32.1%)	50/185 (27.0%)	48/165 (28.9%)
Pain*	96/313 (31.2%)	89/314 (28.3%)	87/312 (28.3%)
Jaundice	10 (3.2%)	2 (0.6%)	2 (0.6%)
Pruritus	1 (0.3%)	3 (1.0%)	3 (0.9%)
Skinfold	None	3 (0.9%)	1 (0.3%)
Skin rash	None	1 (0.3%)	None
Vomiting	143 (45.8%)	153 (48.6%)	164 (52.7%)
Diarrhoea	9 (2.9%)	3 (1.0%)	8 (2.6%
Hepatomegaly	3 (0.9%)	5 (1.6%)	1 (0.3%)
Splenomegaly	31 (9.9%)	34 (10.8%)	32 (10.3%)

Data are presented as mean ± SD for quantitative variables and as patient numbers (%) for categorical variables.

*Information on these self-reported symptoms was not available for young children; the number of patients for whom data was available is thus indicated and used to calculate percentage values.

### Clinical and parasitological responses to treatments

PCR-corrected clinical and parasitological cure rates on Day 28 in the ITT population are shown in Table 4. The two-sided 90% confidence interval of the difference between the ASAQ1 group and the AL group in the proportion of patients with adequate clinical and parasitological response (ACPR) ranged from -0.03 to 0.03. The upper limit of the confidence interval (0.03) was inferior to the pre-specified non-inferiority boundary of 0.05. This indicates that ASAQ1 is not inferior to AL. Similar findings were obtained in the PP population with ACPR for 280 (98.9%) patients in the ASAQ1 group, 285 (100.0%) in the ASAQ2 group and 285 (98.6%) in the AL group.

**Table 4:** PCR-corrected treatment responses in the ITT population on D28.

Outcome	ASAQ1 (N = 310)	ASAQ2 (N = 315)	AL (N = 311)	Total 5
Possible failure (information unavailable)	12 (3.9%)	16 (5.1%)	9 (2.9%)	37 (4.0%)
Late clinical failure	None	None	2 (0.6%)	2 (0.2%)
Late parasitological failure	3 (1.0%)	None	3 (1.0%)	6 (0.6%)
Adequate clinical and parasitological response	295 (95.2%)	299 (94.9%)	297 (95.5%)	891 (95.2%)

Subgroup analyses were performed to assess treatment efficacy in three different age groups (Table 5). The PP analysis confirmed the non-inferiority of the ASAQ1 versus AL treatment group in the <5 year subgroup. The two-sided 90% confidence interval of the difference in response rates between the two treatment groups ranged from -0.0526 to 0.0181. Comparison between the parasitological and clinical cure rate observed in the ASAQ2 group and that of the two other groups demonstrated non-inferiority in the twice daily group. In the ITT population, the two-sided 90% confidence intervals were -0.03 to 0.03 for the difference in therapeutic response rate between ASAQ2 and ASAQ1 and -0.02 to 0.04 for the difference between ASAQ2 and AL.

**Table 5:** PCR-corrected treatment responses in the PP population according to age.

Age group	ASAQ1 (N = 283)	ASAQ2 (N = 285)	AL (N = 289)	Total (N = 857)
< 5 years	132/134 (98.5%)	137/137 (100%)	129/133 (97.0%)	398/404 (98.5%)
5 – 14 years	97/98 (99.0%)	106/106 (100%)	106/106 (100%)	309/310 (99.7%)
≥ 14 years	51/51 (100%)	42/42 (100%)	50/50 (100%)	143/143 (100%)

For all five sites (Table 6), more than 90% of patients showed a PCR-uncorrected ACPR. A number of cases of re-infection were reported, namely one in Cameroon, five in Madagascar, 59 in Mali, and 21 in Senegal (21 in Keur-Socé and none in Mlomp). Regarding the primary endpoint, no centre-related effect was observed.

**Table 6:** Efficacy evaluation on D28 after PCR correction by center (ITT population)

Clinical evaluation	ASAQ1 (N = 310)	ASAQ2 (N = 315)	AL (N = 311)	Total (N = 936)
Cameroon				
Possible failure (information unavailable)	3 (5.6%)	8 (14.5%)	4 (7.1%)	15 (9.1%)
Early treatment failure	0	0	0	0
Late clinical failure	0	0	0	0
Late parasitological failure	0	0	0	0
Adequate clinical and parasitological response	51 (94.4%)	47 (85.5%)	52 (92.9%)	150 (90.9%)

Madagascar				
Possible failure (information unavailable)	0	2 (3.3%)	0	2 (1.1%)
Early treatment failure	0	0	0	0
Late clinical failure	0	0	1 (1.7%)	1 (0.6%)
Late parasitological failure	1 (1.7%)	0	0	1 (0.6%)
Adequate clinical and parasitological response	58 (98.3%)	58 (96.7%)	59 (98.3%)	175 (97.8%)

Mali				
Possible failure (information unavailable)	2 (3.0%)	3 (4.4%)	2 (3.0%)	7 (3.5%)
Early treatment failure	0	0	0	0
Late clinical failure	0	0	0	0
Late parasitological failure	2 (3.0%)	0	2 (3.0%)	4 (2.0%)
Adequate clinical and parasitological response	62 (93.9%)	65 (95.6%)	63 (94.0%)	190 (94.5%)

Senegal – Keur Soce				
Possible failure (information unavailable)	2 (2.1%)	1 (1.1%)	2 (2.2%)	5 (1.8%)
Early treatment failure	0	0	0	0
Late clinical failure	0	0	0	0
Late parasitological failure	0	0	1 (1.1%)	1 (0.4%)
Adequate clinical and parasitological response	93 (97.9%)	91 (98.9%)	86 (96.6%)	270 (97.8%)

Senegal – Mlomp				
Possible failure (information unavailable)	5 (13.9%)	2 (5.0%)	1 (2.6%)	8 (7.0%)
Early treatment failure	0	0	0	0
Late clinical failure	0	0	1 (2.6%)	1 (0.9%)
Late parasitological failure	0	0	0	0
Adequate clinical and parasitological response	31 (86.1%)	38 (95.0%)	37 (94.9%)	106 (92.2%)

### Parasitic clearance and resolution of clinical symptoms

**Figure 2:**
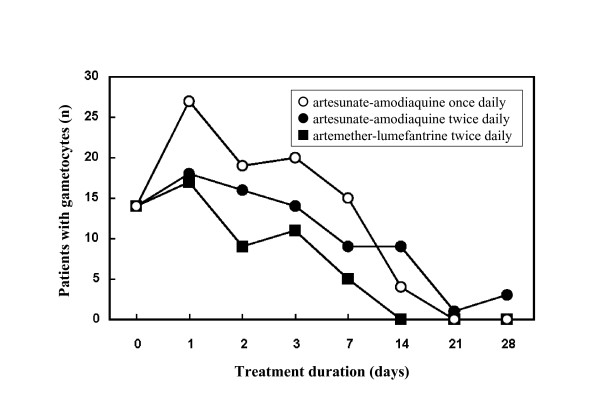
**Gametocyte clearance**. Data are presented as the number of patients with gametocytes.

At enrolment, patients in the three treatment groups had similar mean parasite densities. Parasitic clearance rates were at similar levels at any time and independently of the treatment groups considered: 1.74 ± 0.60; 1.69 ± 0.60 and 1.73 ± 0.59 days in the ASAQ1, ASAQ2 and AL groups respectively (Table 7). The number of gametocyte carriers first increased on Day 1 but dropped from Day 2 in all treatment groups to reach zero on Day 21 in the ASAQ1 group and on Day 14 in the AL group (Figure 2).

**Table 7:** Parasitic clearance

Treatment duration	ASAQ1 (N = 310)	ASAQ2 (N = 315)	AL (N = 311)	Total (N = 936)
Day 0				
N	310 (100%)	315 (100%)	311 (100%)	936 (100%)
Density	37781 ± 45652	41863 ± 49049	33958 ± 41474	37880 ± 45575
Day 1				
N	198 (64.7%)	194 (61.8%)	206 (66.5%)	598 (64.3%)
Density	3297 ± 8403	3137 ± 14753	3764 ± 14809	3406 ± 13002
Day 2				
N	26 (8.6%)	24 (7.7%)	25 (8.1%)	75 (8.1%)
Density	503 ± 787	914 ± 3230	1098 ± 2585	833 ± 2383
Day 3				
N	None	2 (0.6%)	4 (1.3%)	6 (0.7%)
Density	-	154 ± 76	464 ± 296	361 ± 282

Data are presented as the number of patients with parasites (N) and as the mean parasite density ± SD for the patients with parasitaemia.

Rapid clinical improvement was recorded within the three treatment groups. More than 90% of patients had no fever within 24 hours after drug administration. A similar reduction in the prevalence of other symptoms was generally observed as well. Nevertheless, differences between treatment groups for three symptoms were observed only at certain times; vomiting on Day 1 (higher in the ASAQ1 group), asthenia on Day 2 (higher in ASAQ2 group) and headache on Day 3 (higher in the ASAQ1 group).

### Drug safety

Overall, 275 patients (29.3%) in the safety population presented at least one emergent adverse event, of which nature, incidence and intensity were similar between the three treatment groups. Among them, the most frequently reported ones included intestinal parasitic infections (5.4% of all patients; all cases were recorded in Keur-Socé, diagnosed with abdominal symptoms consistent with previous epidemiological studies, but without parasitological confirmation), bronchitis (4.7%) and diarrhoea (3.0%). Investigators considered 20.4% of the emergent adverse events as treatment-related (26.9% in the ASAQ1 group, 20.7% in the ASAQ2 group and 12.3% in the AL group). A significant difference between treatment groups (χ^2 ^test, p = 0.01) was observed in case of mild to moderate somnolence although this was reported only in ASAQ groups in Madagascar (15 children under 14 years of age and two adult patients). Treatment-related adverse events (88.5%) – mainly of mild or moderate intensity – essentially concerned the nervous (24.4%; mostly insomnia and somnolence) and gastrointestinal systems (16.7%). Overall, 122 (13.0%) treated patients experienced vomiting or rejection episodes in the first half-hour after treatment administration from Day 0 to Day 2. No significant difference was recorded between the treatment groups (p = 0.26) although the subgroup including patients under five years of age was significantly more affected by vomiting and rejection. Two cases of rashes reported by investigators as treatment-related were submitted to dermatologists for expertise, with no clear diagnosis established. With respect to clinical symptoms occurring under treatment, a few cases of vomiting were reported in 7.4% of patients, pain in 2.5%, anorexia in 1.8%, pruritus in 1.7%, diarrhoea in 1.3%, asthenia in 0.9%, headache in 0.6% and chills in 0.4%.

No statistical difference was observed between treatment groups in the population presenting abnormal values for haemoglobin, neutrophils, ALT or creatinine. The number of patients with abnormal values for haemoglobin dropped from 50.3% to 32.4% between Day 0 and Day 28. It is worth mentioning that, for all patients whose haemoglobin values were abnormal on Day 28, these values were already abnormal prior to treatment start. The proportion of patients presenting abnormal platelet counts also decreased during the study from 17.7% to 1.1% during the same time lapse. Conversely the percentage of patients with abnormal neutrophil values increased from 1.8% to 8.4%. No statistical difference was observed between treatment groups. Six patients out of seven with severe neutropenia on day 28 kept being monitored after the study completion. In less than four weeks, white blood cell count normalized in all patients. Even though the seventh patient could not be tracked any further due to relocation abroad, signs of well-being could be confirmed through interviews of his relatives still residing on the study site.

The proportion of patients presenting abnormal creatinine values remained stable during the study (20.2% at baseline and 20.4% at D28). For AST and ALT, the proportion of patients with abnormal values decreased during the study from 31.5% on Day 0 to 20.9% on Day 28 for AST, and from 9.2% to 6.0% for ALT. Abnormal values reported for these enzymes were of mild intensity. Except for anemia recorded as an adverse event in seven patients, no abnormal laboratory values were reported as adverse events by the investigators.

Two patients died during the study: one patient in the ASAQ1 group passed away after a lung infection and anemia on Day 3 while one patient in the AL group fell into a fatal coma of unknown cause on Day 1. Investigators did not attribute those deaths to study treatment. One other serious adverse event was reported in the ASAQ1 group, corresponding to a case of severe anemia requiring hospitalization (related to treatment according to the investigator). This event occurred on the fifth day after the last intake of treatment and the patient recovered after hospitalization.

Three patients in the ASAQ1 group discontinued the study due to adverse events (persistence of severe vomiting, fatigue, vertigo and asthenia). These events were considered by the investigator to be severe and probably related to treatment. All patients recovered though.

## Discussion

By demonstrating the non-inferiority of the therapeutic efficacy of ASAQ with respect to AL, this study shows that remarkably accurate clinical and parasitological response can be obtained in patients suffering from uncomplicated *P. falciparum *malaria after treatment with this new formulation. These results are consistent with those from a previous study where AS+AQ were routinely used as multiple tablets (loose combination) in Mali [6], Senegal [19] and in Madagascar [20]. In the per-protocol population the cure rate was >98% in all treatment groups. There was no evidence of clustering of treatment failure in any geographical area and response rates were equivalent in children under five and in older children or adults. Despite of the unavoidable epidemiological versatility related to study sites, such homogenous observations reinforce the confidence in the results depicted in this work.

The two ASAQ1 and ASAQ2 regimens showed similar efficacy and tolerability. This indicates that both regimens are suitable from a clinical point of view, even though ASAQ1 dosing can be expected to improve general compliance thanks to an easier dosage compared to ASAQ2. Considering the present ASAQ packaging, an appropriate dosing may rely on the intake of one tablet per day for children and teenagers, and two tablets once a day for adults. Such a simple administration should help the communities better understand and comply with the treatment.

In agreement with previous studies on AS + AQ mentioned above, all three ACT regimens tested in this work induced a rapid parasitic clearance within three days and a 50% decrease of gametocytaemia in two weeks to reach the complete gametocytes elimination in most of the patients at the end of the study (Day 28). These results confirm those from Sowunmi *et al*, [21] (study on children in Nigeria) that patients treated with AS or AS + AQ had significantly shorter parasite clearance times and lower gametocyte carriage rates than those treated with AQ alone. Those observations as well strongly support that ASAQ can bring the gametocyte carriage rates to a very low level hence contribute to reduce transmission rates of *Plasmodium *sp.

Since fever abated within three days in all but three patients and other malaria symptoms also resolved rapidly in every group, all treatments could be considered well tolerated in terms of safety. They all present a similar clinical and biological safety profile and no unexpected safety issues emerged during the course of the study. Cases of somnolence following ASAQ were reported only in a few children from Madagascar, essentially on Day 0, but this diagnosis relied on (anxious) mother or caretakers facing a "sleepy" episode. Seven cases of severe transient and asymptomatic neutropenia occurred during this study independently of the treatment group. In a cohort of 382 Ugandan patients treated with AS + AQ, AQ + SP or AL, Maiteki-Sebuguzi *et al *[22] reported a patient presenting repeated multiple transient neutropenic episodes, including a severe one after treatment with AS + AQ. In this context, it could be interesting to perform in-depth longitudinal safety monitoring of ASAQ as well as other ACTs with respect to neutropenia evolution throughout treatment courses.

In Africa, the current national policy approach for malaria control is to strengthen the fight through a global plan of elimination, involving reduction of malaria incidence to a very low predetermined level [23]. Besides vector control by using insecticide-treated bed nets and indoor spraying of insecticides, the use of anti-malarial drugs for cure and prevention purposes plays a pivotal role in the fulfillment of this objective. The challenge still relies on the introduction of anti-malarial drugs at the community level outside healthcare facilities since this strategy offers a range of advantages. Firstly, many countries have adopted community case management as a national strategy for malaria control. Secondly, incorporating ACT into community case management has been evaluated in Ghana, Nigeria and Uganda and found to be both feasible and acceptable [24]. It was shown that the success of these programs mainly depends on the presence of well-trained and well-supervised Community Medicine Distributors who will be best suited to manage patients' treatment in villages and provide good therapeutic coverage in an acceptable and proactive way. Finally, in some areas exposed to an epidemic risk of malaria, such as the Highlands of Madagascar [25,26], mass drug administration has been integrated into the campaign to combat malaria epidemics [27]. In these situations, the ASAQ fixed-dose tablets could be a key element in community deployment of ACT. Drug efficacy is a necessary but insufficient requirement to reach these goals. Indeed efforts must be made to make ASAQ affordable and accessible to a wider range of national malaria programs and communities.

## Conclusion

The ASAQ fixed-dose combination administered once daily provided clinical and parasitological efficacy comparable to AL. Non-inferiority of ASAQ with respect to AL was also demonstrated in children under five years of age. Dividing the ASAQ intake into two daily doses does not confer any advantage in terms of efficacy or safety. The ASAQ fixed-dose combination thus offers an effective and safe option to treat uncomplicated *P. falciparum *malaria in adults and children. Effectiveness studies are required now to estimate whether combining a limited number of tablets with a single daily dosage will have a real positive impact on patient compliance. The successful deployment of ASAQ fixed-dose requires operational pharmacovigilance programs which should be conducted to assess its long-term safety profile.

## Competing interests

The authors declare that they have no competing interests. All authors received a stipend from the study sponsor (sanofi-aventis) to cover participation in the study. Valerie Lameyre is employed by sanofi-aventis.

## Authors' contributions

All authors contributed to study design and implementation, patients' inclusion and following-up and data interpretation. All authors read and approved the final manuscript. Especially manuscript revision was due to the commitment of MR, PB and VL. MR and PB are guarantors of the paper.

## Role of the funding source

This study was initiated and funded by sanofi-aventis, manufacturer of Coarsucam™/Artesunate Amodiaquine Winthrop^® ^(artesunate + amodiaquine fixed-dose combination tablets). The sponsor was involved in the study design, the writing of the report, the decision to submit the paper for publication and providing editorial support for the first draft of the manuscript.

## Role of the study supervisory committee

An independent Study Supervisory Committee was established to oversee design and implementation of the study. This committee was consulted before finalizing the protocol and was regularly informed on study progression. It was consulted for notification of adverse events and for classification of protocol deviations. This committee could review any case report forms containing conflicting or questionable efficacy or safety data under blinded conditions. It was consulted for discussion of the results of the study and provided input for finalization of the study report.
